# Supplementary material for: Policy Challenges in Ultra-Rare Cancers: Ethical, Social, and Legal Implications of Melanoma Prevention and Diagnosis in Children, Adolescents, and Young Adults
Source: Healthcare (Basel). 2025 Feb 4;13(3):321. doi: 10.3390/healthcare13030321 (PMC11818049; doi:10.3390/healthcare13030321)
Supplement: Supplementary file 1 [file healthcare-13-00321-s001.zip › healthcare-3421701-supplementary.pdf]

Supplementary Materials

File S1.

Literature review

A. What are the ELSIs associated with prevention and diagnosis of melanoma in childhood?

Research question

The research question was reformulated using the PICO model, which includes the study population (P), the intervention being evaluated (I), the comparator (C), and the outcome of interest (O). Table 1 describes the PICO model underlying this research.

Table S1 – PICO model

**Population**	Children and young adults affected by melanoma
**Intervention(s)**	Prevention and diagnosis
**Comparator(s)**	Not relevant
**Outcome (s)**	Ethical issues
	Legal issues
	Social issues

Search

We searched PubMed/MEDLINE for articles indexed from January 2019 to January 2024, using the search string defined based on the outlined PICO model. We developed a search string comprising three semantic clusters. The first cluster addresses the topics of prevention and diagnosis, the second focuses on melanoma and dermatology, and the third pertains to ethical, social, and legal issues. The complete search string is presented in Table 2.

Table S2 – Search string

((((((diagnosis[Title/Abstract] OR detection[Title/Abstract] OR screening[Title/Abstract] OR prevention[Title/Abstract]) AND (ethic*[Title/Abstract] OR legal[Title/Abstract] OR juridical[Title/Abstract] OR social[Title/Abstract])) AND (skin[Title/Abstract] OR melanoma[Title/Abstract] OR dermatolog*[Title/Abstract]))) NOT ("artificial intelligence"[Title])) NOT (psoriasis[Title]) AND ((y_5[Filter]) AND (fha[Filter]) AND (booksdocs[Filter] OR review[Filter] OR systematic review[Filter]) AND (english[Filter]))) NOT (dermatitis[Title])

Inclusion and exclusion criteria

The documents identified through the search strategy was considered eligible unless it met one or more of the following exclusion criteria:

- duplicate studies;
- studies involving a technology not under investigation;
- studies concerning health conditions not under investigation;
- studies involving a population not under investigation;
- studies that did not fall into the categories of “books and documents,” reviews, or systematic reviews;
- insufficient information reported in the study on any of the investigated aspects;
- abstract/full text not available;

- study not available in English.

The reference lists of the included documents were manually checked for additional relevant studies. We did not assess the methodological quality of the included studies.

Study selection

The Table 3 details the study selection.

Table S3 – Study selection

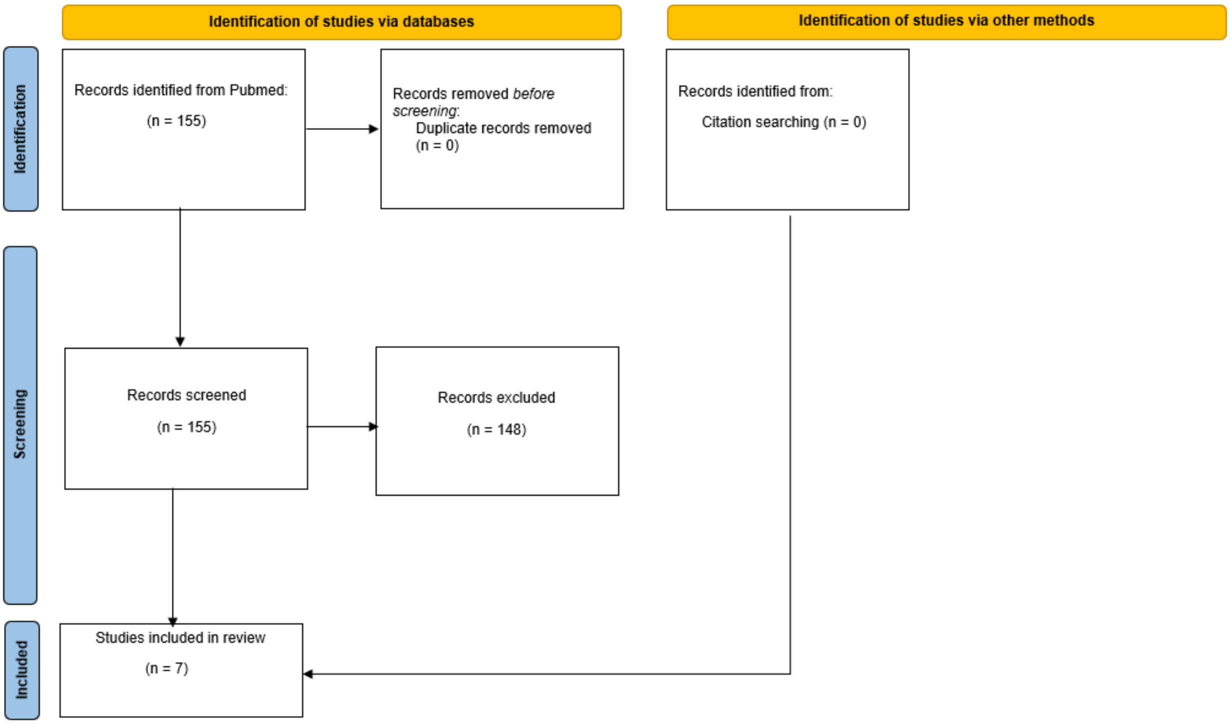

Data extraction

The Table 4 details the data extraction.

Table S4 – Data extraction

**Source**	**Type of practice(s)**	**Focus**	**Type of participant(s)**	**General considerations**	**Ethical issues**	**Legal issues**	**Social issues**
Glines KR, Haidari W, Ramani L, Akkurt ZM, Feldman SR. Digital future of dermatology. Dermatol Online J. 2020;26(10):13030/qt75p7q57j.	Imaging technologies in the field of dermatology	Teledermatology: status and future, challenges and opportunities	Unspecified	Patients and healthcare providers are satisfied with teledermatology as they are able to experience shorter waiting times and decreased costs. It is important to consider the new ethical and legal limitations of teledermatology, such as changes in documentation, patient privacy, and reimbursement models	-equality in terms of access<br>- improves diagnostic accuracy	-unique issues of privacy, liability and security related to apps require an updated legal framework<br>-reimbursement issues<br>-clinical records and documentation	-underserved communities and those in rural settings are more likely to have a dermatologic evaluation by a specialist via teledermatology<br>- shorter waiting time<br>- lower costs
Kashetsky N, Mar K, Liu C, Rivers JK, Mukovozov I. Photography in dermatology - a scoping review: Practices, skin of color, patient preferences, and medical-legal considerations. *J Dtsch Dermatol Ges.* 2023;21(10):1102-1107. doi:10.1111/ddg.15129	Clinical photography in dermatology	Analysing the spreading use of clinical photography in dermatology	Unspecified	Photography is commonly used in biopsy site marking, assessment and diagnosis, disease monitoring, treatment response, medical education, research, seeking advice from colleagues, and tele dermatology. Most patients are supportive but there are some challenges to look at	-informed consent	-informed consent must always be clear and written<br>-where images are stored and how they are shared<br>- privacy	
Lei BUW, Prow TW. A review of microsampling techniques and their social impact. *Biomed Microdevices.* 2019;21(4):81. doi:10.1007/s10544-019-0412-y	Diagnosis (of melanoma among others)	Microsampling techniques for disease diagnosis	Unspecified	Microsampling techniques are less invasive than conventional skin (and blood) sampling. More attention is given to the patient's individual feelings. Microsampling may help develop a decentralized diagnostic model (vs centralized model).	-less invasive than conventional sampling methods<br>-cheaper than conventional sampling methods<br>-decentralized diagnostic model will lead to clinical decisions being taken in a timely manner, and diagnostic procedures being more accessible to patients<br>-all these benefits may lead to an improvement in treatment outcomes especially for patients who are in remote areas or require regular diagnostic services		-on a social level, microsampling aligns with the demand for a patient-centric diagnostic experience<br>-this technique will help those patients who are in more remote areas
Li HO, Bailey AJ, Grose E, et al. Socioeconomic Status and Melanoma in Canada: A Systematic Review. *J Cutan Med Surg.* 2021;25(1):87-94. doi:10.1177/1203475420960426	Relevance of socioeconomic status and melanoma diagnosis	Fills the gap in the literature by performing a systematic review pertaining Canada, a country with a universal healthcare system	Unspecified	Positive association between melanoma incidence and high socioeconomic status (SES).Tthe review also highlights the association between low SES and late-stage melanoma at presentation. This echoes what found in a review conducted in Northern Europe: associations between high SES and increased melanoma risk, thinner tumors, increased survival, and decreased mortality. The hypothesis is that due to increased accessibility to screening, the higher SES population experiences more early detection - perhaps incurring the problem of overdiagnosis too?	-inequality, great potential for disparities in access<br>-overdiagnosis is already a problem in itself, and it also causes undertreatment for those who have less access		-health disparities determined also by social status make the social status be perceived even more as a burden
Najmi M, Brown AE, Harrington SR, Farris D, Sepulveda S, Nelson KC. A systematic review and synthesis of qualitative and quantitative studies evaluating provider, patient, and health care system-related barriers to diagnostic skin cancer examinations. *Arch Dermatol Res.* 2022;314(4):329-340. doi:10.1007/s00403-021-02224-z	Diagnosis of melanoma	Identifies barriers for skin screening by providers (primary care physicians and advanced care physicians), patients and health systems	Unspecified	Future melanoma detection initiatives must be structured based on a firm understanding of the barriers and challenges encountered by providers, patients and public health systems	- public surveillance programs are resources intensive (this can be a problem for public health)<br>-poor access (can be a problem from the patient side)<br>-costs/fear (patients side)<br>-lack of access to knowledge and skills (physicians side);<br>-no clarity about what to do (physicians side)<br>-access to specialized care can be difficult in certain areas<br>-misdiagnosis and overdiagnosis	-inadequate reimbursement can be a barrier	-social prejudice leading to tanning booths, lack of adequate public awareness (public health side)<br>-lack of knowledge, lack of comfort (patients side)
Ploderer B, Rezaei Aghdam A, Burns K. Patient-Generated Health Photos and Videos Across Health and Well-being Contexts: Scoping Review. *J Med Internet Res.* 2022;24(4):e28867. doi:10.2196/28867	Use of photos and videos in health context	Identifies the key themes in patients' use of health photos and videos: values gained by patients as well as challenges experiences	Unspecified	The article expands on the use of photos and videos in health contexts, including melanoma detection. While such practices can help patients and even foster education among patients, it is not without challenges	-challenges associated with taking pictures: accessibility, incomplete image sets, and image quality<br>-challenges associated with image sharing: harmful feedback, misinformation<br>-challenges associated with image examination: interpretability, with the risk of overestimating the relevance of a skin lesions, or missing a diagnosis	-challenges associated with image sharing: privacy	-emotional labor
Sadrolashrafi K, Cotter DG. Not Your Mother's Melanoma: Causes and Effects of Early Melanoma Diagnosis. *Dermatopathology (Basel).* 2022;9(4):368-378. doi:10.3390/dermatopathology9040043	Early diagnosis of melanoma	The problem of overdiagnosis of melanoma, with its limitations and challenges	Unspecified	The melanoma epidemic is a phenomenon heavily influenced by current pressures to not miss melanoma and to diagnose melanoma as early as possible – two goals of extreme merit that inadvertently result in overtreatment of many lesions due to the limitations of current staging guidelines for CM	-not beneficial to the good of patients (overtreating innocuous melanomas and may also undertreat aggressive melanomas)<br>-not beneficial to the good of healthcare systems<br>-healthcare providers practicing defensive medicine for fear (lowering threshold)<br>-inequality in treating different age groups	-defensive medicine	- overdiagnosis and overtreatment may generate a vicious cycle that worsens the patient's quality of life for the psychological and financial tool that they cause

File S2.

B. What are the ELSIs in the context of prevention and diagnosis of (ultra)rare diseases?

Research question

The research question was reformulated using the PICO model. Table 5 describes the PICO model underlying this research.

Table S5 – PICO model

**Population**	Persons affected by (ultra)rare diseases
**Intervention(s)**	Prevention and diagnosis
**Comparator(s)**	Not relevant
**Outcome (s)**	Ethical issues
	Legal issues
	Social issues

Search

We searched PubMed/MEDLINE for articles indexed from January 2019 to January 2024, using the search string defined based on the outlined PICO model. We developed a search string comprising three semantic clusters. The first cluster addresses the topic of rare diseases, the second focuses on prevention and diagnosis, and the third pertains to ethical, social, and legal issues. The full search string is provided in Table 6.

Table S6 – Search string

(("rare disease") AND (diagnosis[Title/Abstract] OR detection[Title/Abstract] OR screening[Title/Abstract] OR prevention[Title/Abstract])) AND (ethic*[Title/Abstract] OR legal[Title/Abstract] OR juridica[Title/Abstract] OR social[Title/Abstract])

Inclusion and exclusion criteria

The documents identified through the search strategy was considered eligible unless it met one or more of the following exclusion criteria:

- duplicate studies;
- studies involving a technology not under investigation;
- studies concerning health conditions not under investigation;
- studies involving a population not under investigation;
- studies that did not fall into the categories of “books and documents,” reviews, or systematic reviews;
- insufficient information reported in the study on any of the investigated aspects;
- abstract/full text not available;
- study not available in English.

The reference lists of the included documents were manually checked for additional relevant studies. We did not assess the methodological quality of the included studies.

Study selection

The Table 7 details the study selection.

Table S7 – Study selection

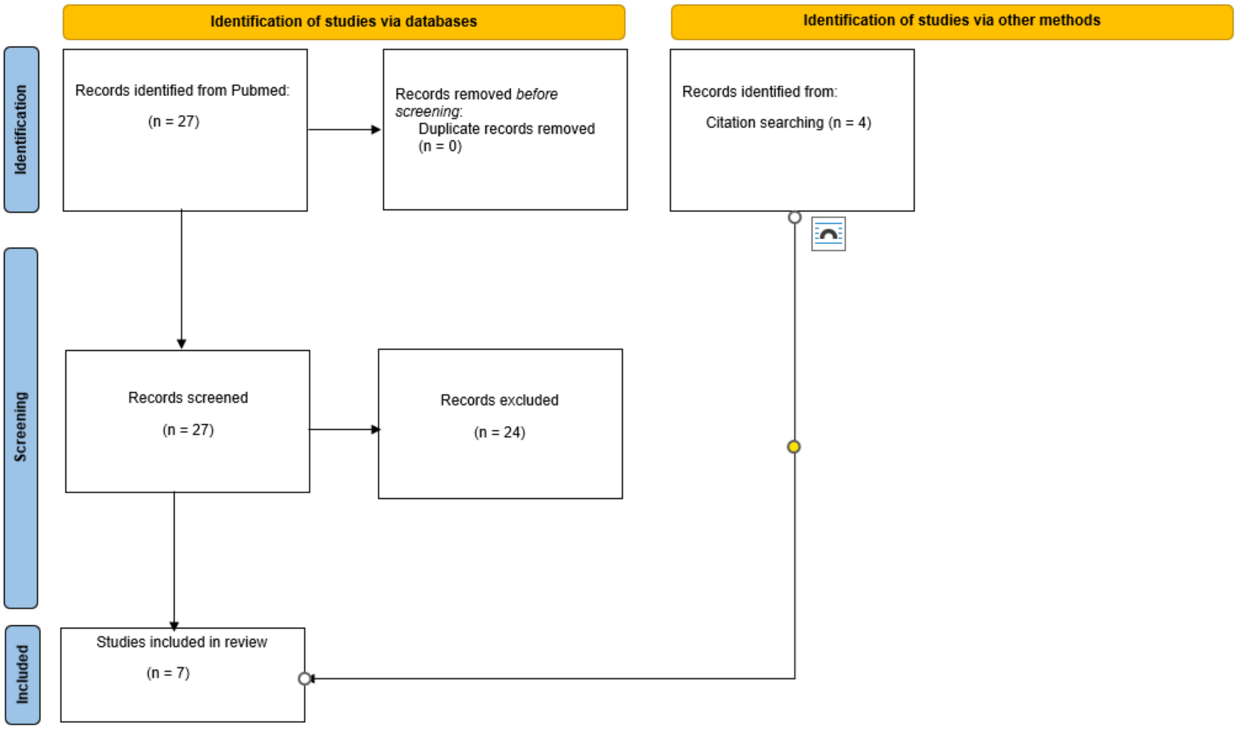

Data extraction

The Table 8 details the data extraction.

Table S8 – Data extraction

Source	Type of practice(s)	Focus	Type of participant(s)	General considerations	Ethical issues	Legal issues	Social issues
Abdallah S, Sharifa M, I Kh Almadhoun MK, et al. The Impact of Artificial Intelligence on Optimizing Diagnosis and Treatment Plans for Rare Genetic Disorders. *Cureus*. 2023;15(10):e46860. doi:10.7759/cureus.46860	Diagnosis and treatment for rare genetic disorder	Evaluates the impact of AI and ML (Machine Learning) in addressing the challenges posed by rare genetic disorders	Unspecified	AI and ML are reshaping medical decision-making through their capacity to harness extensive datasets and computational capabilities.<br>Their usage holds the promise of not only improving patient outcomes but also alleviating the burden on healthcare systems by streamlining processes and enhancing overall efficiency	-issues regarding privacy and data ownership<br>-equity and management of personal health data and potential biases inherent in AI systems<br>-equally problematic: the omission of rare diseases from AI training datasets which may be detrimental and generate biases precluding equitable representation<br>-another point to be addressed is the management of secondary findings for patients as well as relatives	-poor global AI regulations in terms of clear responsibilities within AI healthcare application<br>-issues of security to preclude data breaches; safeguarding human genetic resources; precluding misappropriation of genetic information. -consent form must explicitly elucidate the purposes of data utilization, providing a robust legal shield<br>-also to be addressed: issue of consent concerning the disclosure of incidental findings by AI systems	-equitable delivery of healthcare services given the biases inherent in AI algorithms<br>-job losses as AI adoption escalates
Adachi T, El-Hattab AW, Jain R, et al. Enhancing Equitable Access to Rare Disease Diagnosis and Treatment around the World: A Review of Evidence, Policies, and Challenges. *Int J Environ Res Public Health*. 2023;20(6):4732. doi:10.3390/ijerph20064732	Diagnosis and treatment for RDs	Provides a comprehensive summary of evidence on the current situation of RDs globally and regionally, including current conditions, practices, policies, and regulations, as well as the challenges and barriers faced by RD patients and their families and caregivers	Unspecified	RDs have profound economic and societal costs. Yet, responding to the needs of the RD community is the only thing to do if one wants to promote health equity. The review ends with a long list of recommendations for countries, stakeholders, and decision makers	-inequality: uneven access to orphan medicines, which, in turn, affects the quality of care and treatment that RD patients receive<br>-late diagnosis which leads to poor disease management<br>-limited research and development also due to the heterogeneity of the RD population (and the fact that a great percentage of them are minor) and limited funding	-issues with reimbursement and access to orphan drugs	-social and cultural exclusion, limited political visibility and limited prioritization<br>-difficulties accessing adequate diagnosis, treatment, and care<br>-heavy emotional, financial, and social burdens<br>-struggle to navigate the healthcare system<br>-huge disparities in RD quality of care and access to treatment among different countries
Esquivel-Sada D, Nguyen MT. Diagnosis of rare diseases under focus: impacts for Canadian patients. *J Community Genet*. 2018;9(1):37-50. doi:10.1007/s12687-017-0320-x	Diagnosing RDs	Grasps the role of receiving a diagnosis for patients with RDs	Unspecified	The study offers an empirical analysis of the impact of receiving or not receiving a diagnosis for RD patients: positive outcomes (in terms of treatment, as well as self-confidence and validation) as well as challenges (discrimination, stigma, insurance discrimination and right not to know)	-ethical justification of testing when there is no therapeutic benefit / testing used for information only<br>- testing when there is no therapeutic benefit: individual vs collective interest<br>-equality<br>-incidental findings for patients as well as relatives	-raises the issue of legally protecting those with a RD diagnosis from insurance discrimination and discrimination in the workplace with legislations	-access to additional services and subsidies<br>-insurance discrimination<br>-self-confidence<br>-validation<br>-life planning and reproductive decision making
Gainotti S, Mascalzoni D, Bros-Facer V, et al. Meeting Patients' Right to the Correct Diagnosis: Ongoing International Initiatives on Undiagnosed Rare Diseases and Ethical and Social Issues. *Int J Environ Res Public Health*. 2018;15(10):2072. doi:10.3390/ijerph15102072	Diagnosis of RDs	Focuses on the need and right for patients to receive a diagnosis, which necessarily involves a more engaged and better managed relationship between patients and doctors, as well as between patient community and research community	Unspecified	The social, ethical and legal issues at stake for a patient may be quite different from how one would perceive from the outside. It is important to engage with patient and let them collaborate throughout the process of diagnosis, with a focus on data sharing	-privacy: struggling to find a balance between excessive paternalism (anonymity may not be the patient's first concern) and undue liberalism (protecting confidentiality is still relevant)<br>-patients literacy through genetic counselling about the findings they will be exposed to<br>-especially important patient involvement in the communication of secondary findings<br>-informed consent<br>-cost/benefit balance (also note: cost of storing more and more data as more and more genetic screenings are performed)	-risk of personal data leakage, misuse of data, direct use of data for purposes not related with the aim of research, re-identifications<br>-informed consent and indirect diagnosis of family members and children, invasion of the right (not) to know	-doctor-patient relationship and communication: patients struggle as they are often dismissed by doctors<br>-patients literacy
Groft SC, Posada M, Taruscio D. Progress, challenges and global approaches to rare diseases. *Acta Paediatr*. 2021;110(10):2711-2716. doi:10.1111/apa.15974	Diagnosis and disease management of RDs	Reviews the status of rare diseases: progress and challenges	Unspecified	RDs present a challenge for diagnosis and treatment to all clinicians and healthcare practitioners.<br>Better diagnostic criteria procedures and improved treatments are now available for more RDs as approved or investigational products.<br>Patient-centered approaches are becoming more common in the care of patients and families through novel communication media offered by telehealth and telemedicine	-disease management efforts at local and regional levels are undermined.<br>-since over half of the population affected by RDs are children, special ethical considerations limit the possibility to enroll this population in clinical trials, which can slow down the development of new therapies.<br>-it is important to standardize RD research approach and technology to improve the chances to compare data	-accessibility is also deeply affected by reimbursement policies and access to orphan drugs	-great disparities in gaining access to a correct diagnosis and access to care due to geographic location and socioeconomic status of patients exist in many countries<br>-stigmatization<br>-importance to be represented by Patient Advocacy Groups that can build partnerships with professionals and societies
Hallowell N, Badger S, McKay F, Kerasidou A, Nellåker C. Democratising or disrupting diagnosis? Ethical issues raised by the use of AI tools for rare disease diagnosis. *SSM Qual Res Health*. 2023;3:100240. doi:10.1016/j.ssmqr.2023.100240	Using AI tool for RD diagnosis	Assesses whether the use of computational phenotyping (CP) technology which uses facial recognition algorithms to classify and potentially diagnose rare genetic disorders can have benefits	Unspecified	While there is widespread agreement among stakeholders regarding the public benefits of AI assisted diagnosis, there are also concerns about it	-fosters equality among patients, particularly in low- and middle-income countries and rural regions<br>-algorithm bias could be a problem: the AI still must be trained and supervised<br>-grater diagnostic confidence and save time	-liability	-such tool could upskill non-clinicians but on the other hand also deskill the experts<br>-it may encourage self-diagnose which is a big problem and a generator of anxiety<br>-it could dehumanize medical act
Kruse J, Mueller R, Aghdassi AA, Lerch MM, Salloch S. Genetic Testing for Rare Diseases: A Systematic Review of Ethical Aspects. *Front Genet*. 2022;12:701988. doi:10.3389/fgene.2021.701988	Genetic testing for RDs	Presents a detailed overview of ethical aspects relevant to genetic testing for RDs	Unspecified	The review found that not many physicians find themselves in a position where they feel knowledgeable enough to order ad conduct genetic testing, especially for rare diseases. As this knowledge and comprehension should be implemented, education should include also the ethical and legal issues presented in the review	-consent: right to know/ right not to know<br>-incidental findings that may affect family members (right to know/right not to know)<br>-ethical justification of testing when there is no therapeutic benefit / genetic testing used for information only<br>-privacy concerns<br>-costs of testing<br>-storage of huge amount of data	-data security<br>-regulation to prevent discrimination in the workplace from happening	-stigma and discrimination<br>(in the workplace, or for family planning decisions)<br>-diagnosis odyssey<br>-increased pressure to test

File S3.

C. What are the ELSIs associated with the use of AI for prevention and diagnosis in dermatology?

Research question

The research question was reformulated using the PICO model. Table 10 describes the PICO model underlying this research.

Table S9 – PICO model

**Population**	Persons affected by dermatological diseases
**Intervention(s)**	AI-driven technologies for detection
**Comparator(s)**	Not relevant
**Outcome (s)**	Ethical issues
	Legal issues
	Social issues

Search

We searched PubMed/MEDLINE for articles indexed from January 2019 to January 2024, using the search string defined based on the outlined PICO model. We developed a search string comprising three semantic clusters. The first cluster addresses the topic of artificial intelligence, the second focuses on prevention and diagnosis, and the third pertains to ethical, social, and legal issues. The full search string is provided in Table 10.

Table S10 – Search string

((("artificial intelligence"[Title/Abstract] OR algorithm*[Title/Abstract] OR "machine learning"[Title/Abstract] OR "deep learning"[Title/Abstract]) AND (diagnosis[Title/Abstract] OR detection[Title/Abstract] OR screening[Title/Abstract] OR prevention[Title/Abstract])) AND (skin[Title/Abstract] OR melanoma[Title/Abstract] OR dermatolog*[Title/Abstract])) AND (ethic*[Title/Abstract] OR social[Title/Abstract] OR legal[Title/Abstract] OR juridical[Title/Abstract])

Inclusion and exclusion criteria

The documents identified through the search strategy was considered eligible unless it met one or more of the following exclusion criteria:

- duplicate studies;
- studies involving a technology not under investigation;
- studies concerning health conditions not under investigation;
- studies involving a population not under investigation;
- studies that did not fall into the categories of "books and documents," reviews, or systematic reviews;
- insufficient information reported in the study on any of the investigated aspects;
- abstract/full-text not available;
- study not available in English.

The reference lists of the included documents were manually checked for additional relevant studies. We did not assess the methodological quality of the included studies.

Study selection

The Table 11 details the study selection.

Table S11 – Study selection

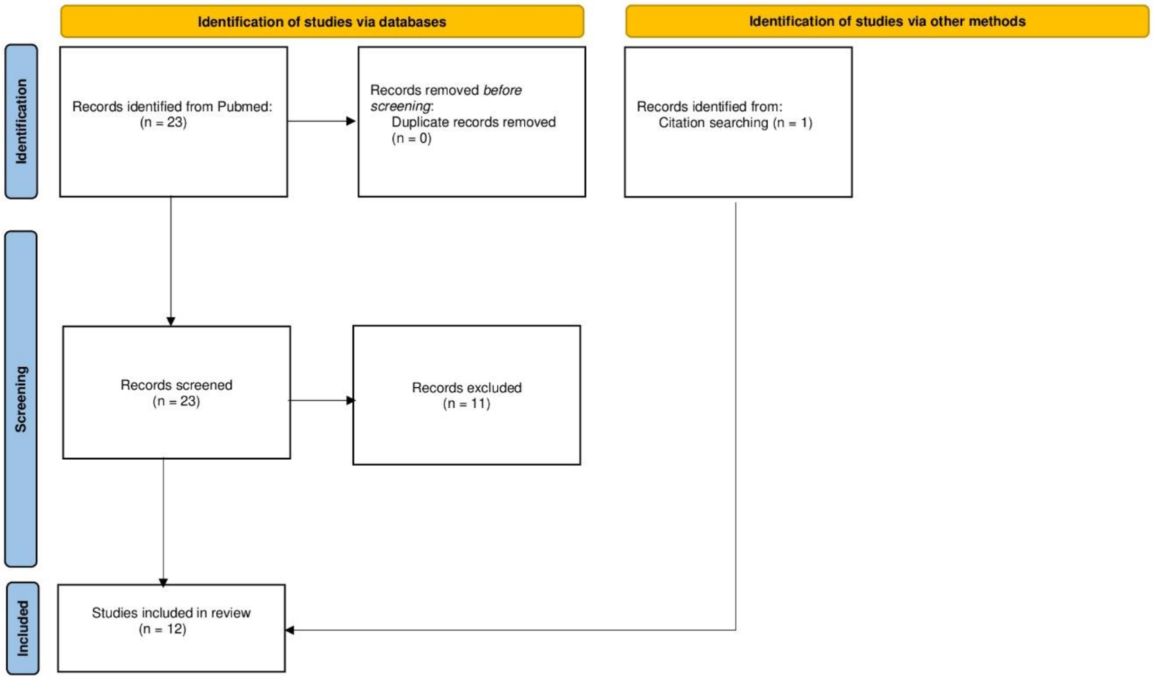

Data extraction

The Table 12 details the data extraction.

Table S12 – Data extraction

Source	Type of practice(s)	Focus	Type of participant(s)	General considerations	Ethical issues	Legal issues	Social issues
Chu YS, An HG, Oh BH, Yang S. Artificial Intelligence in Cutaneous Oncology. Front Med (Lausanne). 2020 Jul 10;7:318. doi: 10.3389/fmed.2020.00318	Diagnosis of skin cancer	Decisions regarding whether or not to conduct a biopsy	Unspecified	Inaccuracy of the available technology	- to form a consensus between the patient and the doctor<br>- unnecessary stress on the user<br>- unnecessary visits to the dermatologist<br>- many apps require users to agree to their data policies, but the methods of mining, using, and sharing patient data are often not transparent<br>- it is unclear how responsibilities for medical malpractice will be determined if a patient is injured due to inaccurate information	- to prevent third parties accessing data during the transmission process (legal liability)<br>- to solve this problem, a supervisory institution in which doctors participate is required<br>-to focus on securing high-quality data	
Daneshjou R, Smith MP, Sun MD, Rotemberg V, Zou J. Lack of Transparency and Potential Bias in Artificial Intelligence Data Sets and Algorithms: A Scoping Review. JAMA Dermatol. 2021 Nov 1;157(11):1362-1369. doi: 10.1001/jamadermatol.2021.3129.	Diagnosis of skin cancer	To assess whether data sets used for training diagnostic AI algorithms addressing skin disease are adequately described and to identify potential sources of bias in these data sets	Unspecified	3 issues in data sets that are used to develop and test clinical AI algorithms for skin disease that should be addressed before clinical translation:<br>1. sparsity of data set characterization and lack of transparency<br>2. nonstandard and unverified disease labels<br>3. inability to fully assess patient diversity used for algorithm development and testing			
Daneshjou R, Barata C, Betz-Stablein B, Celebi ME, Codella N, Combalia M, Guitera P, Gutman D, Halpern A, Helba B, Kittler H, Kose K, Liopyris K, Malvehy J, Seog HS, Soyer HP, Tkaczyk ER, Tschandl P, Rotemberg V. Checklist for Evaluation of Image-Based Artificial Intelligence Reports in Dermatology: CLEAR Derm Consensus Guidelines From the International Skin Imaging Collaboration Artificial Intelligence Working Group. JAMA Dermatol. 2022 Jan 1;158(1):90-96	To guide developers and reviewers of dermatology AI	To develop image-based algorithms for dermatology applications	Unspecified	A lack of standardization among imaging modalities and the risk of bias from noisy labels or demographically unrepresentative data		- all information should be in alignment with legal/privacy data protection, and be addressed with appropriate consents to permit openness and scientific rigor<br>- this recommendation is also relevant for a regulatory framework and should be considered for any automated dermatology algorithm that may affect the wider community	
Du-Harpur X, Watt FM, Luscombe NM, Lynch MD. What is AI? Applications of artificial intelligence to dermatology. Br J Dermatol. 2020 Sep;183(3):423-430. doi: 10.1111/bjd.18880.	Applications of AIto dermatology	equip clinicians with the relevant knowledge to critically appraise future studies, and also assess the clinical utility of this technology	Unspecified	- generalizability: as algorithms are fundamentally a reflection of their training data, this means that if the input image dataset is biased in some way, this will have a direct impact on algorithmic performance<br>- in order to capture more melanomas (increased sensitivity), the algorithm may incorrectly misclassify more benign naevi as malignant (false-positives): this could potentially lead to unnecessary biopsies for patients, which aside from patient harm would create additional demand on an already burdened healthcare system<br>- AI technologies are being deployed in healthcare in two main scenarios: direct to consumer and as a decision aid for clinicians. The benefits and risks, such as false reassurance and unnecessary referrals, are still unclear. While health data democratization supports patient autonomy, it may not necessarily lead to better health outcomes and could result in unnecessary concern and investigations	Safety Perspective Considerations:<br>- Algorithm Standards: To replace a dermatologist, an algorithm must match the current gold standard of clinical assessment by a dermatologist, who examines the lesion considering patient history and the rest of their skin.<br>- Comparative Studies: Published studies compare neural networks with dermatologists using dermoscopic or clinical images, often with limited additional clinical information, rather than the comprehensive clinical assessment standard.<br>- Training Data Limitations: Training data lack sufficient quantities of certain lesion types, especially rarer malignancies like amelanotic melanoma, raising concerns about algorithm performance with novel, potentially malignant lesions	- lack of explainability by neural networks: The European Union's General Data Protection Requirement specifies explainability as a requirement for algorithmic decision making, which is currently not achievable<br>- healthcare is currently structured in such a way that responsibility and liability are carried by the provider and not the patient, and as such these apps do not have a clear-cut position in healthcare infrastructure	
Jobson D, Mar V, Freckelton I. Legal and ethical considerations of artificial intelligence in skin cancer diagnosis. Australas J Dermatol. 2022 Feb;63(1):e1-e5. doi: 10.1111/ajd.13690	AI in skin cancer diagnosis	Legal and ethical issues	Unspecified		- the rate of false negatives, or sensitivity, as there is a potential for inappropriate reassurance<br>- overdiagnosis of melanoma<br>- financial burden to patients and the health system<br>- Another danger is uncritically relying on AI for diagnosis and treatment, which may lack necessary clinical nuance. This issue is also known as 'atrophy of vigilance' in risk evaluation contexts.- Privacy<br>- de-identification of photographic data is another privacy issue	- who has a duty of care to the users and is legally liable for AI errors?<br>- the patient should give consent to the use of AI software	
Jones OT, Matin RN, van der Schaar M, Prathivadi Bhayankaram K, Ranmuthu CKI, Islam MS, Behiyat D, Boscott R, Calanzani N, Emery J, Williams HC, Walter FM. Artificial intelligence and machine learning algorithms for early detection of skin cancer in community and primary care settings: a systematic review. Lancet Digit Health. 2022 Jun;4(6):e466-e476. doi: 10.1016/S2589-7500(22)00023-1	Proposed checklist for the design, development, and evaluation of artificial intelligence and machine learning (AI/ML) algorithms aiming to support the triage or detection of possible skin cancers		Unspecified	The design, development, and evaluation of AI/ML algorithms for skin cancer triage or detection require a structured and standardized approach to ensure reliability, accuracy, and clinical applicability			

Kashetsky N, Mar K, Liu C, Rivers JK, Mukovozov I. Photography in dermatology - a scoping review: Practices, skin of color, patient preferences, and medical-legal considerations. J Dtsch Dermatol Ges. 2023 Oct;21(10):1102-1107	Photography in dermatology	Practices, skin of color, patient preferences, and medical-legal considerations	Unspecified	Patient preference in photography in clinical dermatology		Pertinent medical-legal issues include concerns around privacy, personal device use, and documentation of consent	
Muralidharan V, Burgart A, Daneshjou R, Rose S. Recommendations for the use of pediatric data in artificial intelligence and machine learning ACCEPT-AI. NPJ Digit Med. 2023 Sep 6;6(1):166. doi: 10.1038/s41746-023-00898-5.	Use of data in AI and ML	Pediatric data	Pediatric	Sources of age related algorithmic bias:<br>- lack of age reporting<br>- lack of representation<br>- inappropriate<br>- generalizations made to pediatric populations from adult data<br>- inappropriate -generalizations made to adult populations from pediatric data.<br>- Unique characteristics of pediatric data:<br>- physiologically and anatomically different from adults<br>- heterogeneity in developmental stages within pediatric population<br>- protected research population<br>- unique ethical considerations at each developmental stage	- Age<br>- Communication<br>- Consent and assent<br>- Equity<br>- Data protection<br>- Technological considerations - (transparency of techniques training, and testing methodology)	- Differentiating de-identifiable and identifiable data is a key consideration for safe data regulation, as legislation surrounding consent and data protection differ for the respective categories<br>- While the development of specific laws that are tailored to pediatric data usage may be beneficial, existing legal processes must be optimized for transparency with both pediatric subjects, their parents or legal guardians	
Pai VV, Pai RB. Artificial intelligence in dermatology and healthcare: An overview. Indian J Dermatol Venereol Leprol. 2021 [SEASON];87(4):457-467. doi: 10.25259/IJDVL_518_19	AI in dermatology	Not specified	Not specified	Data: Artificial intelligence applications are only as robust as the data on which they are trained. Deep learning neural networks require large amounts of data. This can be a drawback when artificial intelligence is attempted on a disease with low prevalence or when data is generalised across different populations<br>Acceptance: patient care is not restricted to diagnosis but requires a holistic approach with human touch which cannot be replaced by algorithm<br>The black box problem: it thus becomes difficult to determine which specific feature or calculation of the input data contributed to the resultant output<br>Bias: Algorithms may also inherit the bias of the programmer or the self-learning algorithms may learn to be biased due to lack of diversity in the training material<br>Job threat: There is a huge debate among experts whether or not artificial intelligence will result in large scale job losses	The efficacy of the algorithms depends on large data and certain data may infringe on patients' privacy. Therefore, it is important to create standard ethical guidelines wherein artificial intelligence can be applied and where they are mandatory. Also, in the case of an adverse event, accountability is still a grey area that has to be addressed		
Phung M, Muralidharan V, Rotemberg V, Novoa RA, Chiou AS, Sadée CY, Rapaport B, Yekrang K, Bitz J, Gevaert O, Ko JM, Daneshjou R. Best Practices for Clinical Skin Image Acquisition in Translational Artificial Intelligence Research. J Invest Dermatol. 2023 Jul;143(7):1127-1132. doi: 10.1016/j.jid.2023.02.035.	Clinical skin image acquisition in translational AI research	Best practices	Unspecified			- regulations for translational AI studies in dermatology differ depending on the jurisdiction in which the study is taking place and the method of image and data acquisition<br>- key image acquisition guidelines include avoiding the use of surgical skin markers or rulers to minimize bias in training, reducing background noise, avoiding flash when photographing skin of color, and ensuring quality improvement processes are in place.<br>- image labeling methods should be carefully considered and justified, as limitations exist for both "gold standard" histopathology and clinical labeling, potentially leading to imperfect ground truth when training a machine learning model.<br>- before data collection, the study team (consisting of clinical and technical personnel) should clearly outline the intended clinical purpose, use, and hypotheses that might be tested with the dataset and communicate regularly to ensure	

						that the dataset is optimized for machine learning	
Sengupta D. Artificial Intelligence in Diagnostic Dermatology: Challenges and the Way Forward. Indian Dermatol Online J. 2023 Oct 17;14(6):782-787. doi: 10.4103/idoj.idoj_462_23	Current state of AI in diagnostic dermatology	Challenges	Unspecifed		- data quality and quantity<br>- algorithm development and explainability		- trustis currently lacking among both dermatologists (national survey) and the general population
Young AT, Xiong M, Pfau J, Keiser MJ, Wei ML. Artificial Intelligence in Dermatology: A Primer. J Invest Dermatol. 2020 Aug;140(8):1504-1512. doi: 10.1016/j.jid.2020.02.026.	Diagnosis of skin lesions	1. teledermatology, including triage for referral to dermatologists<br>2. augmenting clinical assessment during face-to-face visits<br>3 dermatopathology	Unspecified	- Adequate representation of underserved populations in training data is essential: existing deep learning models have predominantly been trained on European or East Asian populations, and the relative lack of training on darker skin pigmentation may limit overall diagnostic accuracy<br>- Poor quality images are often excluded from studies, but the problem of what makes an image "adequate" is not well studied<br>- It is difficult to study generalizability because published deep learning models are not publicly available, making it impossible to compare performance<br>-There are significant barriers to implementing AI, with technical considerations including model generalizability, confidence calibration, and interpretability	- AI has the potential to worsen healthcare disparities<br>- AI may perpetuate inadvertent discriminatory practices, for example in recommending less follow-up for dark-skinned patients<br>- acceptance of AI in clinical decision-making depends on understanding the decision-making process behind its predictions. Deep learning models are inherently difficult to interpret due to their complexity and millions of learned parameters, making interpretation of their output an active field of research	- defending against security threats, and navigating the regulatory landscape<br>- how AI technologies will be reimbursed is unclear	- access to, and use of technology differs based on sociodemographics, and more tech-savvy users may be more likely to embrace AI for skin screening

File S4.

FOCUS GROUP 1

March 23, 2024, Berlin

PARTICIPANTS: 15 members + 2 moderators + 2 rapporteurs

15 members:

- 3 patients with young adult melanoma
- 2 patients with adult melanoma
- 3 caregivers - 1 young adult melanoma and 2 adult melanoma
- 2 caregivers of children diagnosed with melanoma
- 1 caregiver of an adolescent diagnosed with melanoma
- 2 CNM patients – a young adult patient and a caregiver
- 1 other type of rare melanoma (uveal)
- 1 nurse (melanoma trained)

Participants country of origin, knowledge, and experiences: Netherlands (5), Romania (4), Sweden (2), Poland (1), Germany (1), Denmark (1) and Spain (1). The experiences are mixed as people have patient and patient advocacy experiences in more than one country. About half of 15 participant are patient advocates and 6 are beginners or looking forward to help in patient advocacy

2 moderators:

- Laura Sampietro-Colom; Clinic Barcelona University Hospital / Clinic Barcelona Research Foundation
- Pietro Refolo; Università Cattolica del Sacro Cuore

1 rapporteur:

- Costanza Raimondi; Università Cattolica del Sacro Cuore

Workshop facilitated thanks to MELCAYA project partner AMeR (The Romanian Melanoma Association) in collaboration with MPNE (Melanoma Patient Network Europe).

NOTE*: The content of this document reflects individual opinions and personal experiences shared by participants who are directly or indirectly affected by melanoma. It does not represent consensus views or official recommendations from the MPNE and AMeR.*

DURATION: 2 hours

Table S13. Ethical, Legal and Social Implications of diagnosis and prevention of childhood melanoma: issues found in the literature were presented, and participants of the Focus Group (1) added their inputs. Only those issues on which the participants added something are reported in the results.

Issues found in the literature	Inputs from participants
Lack of adequate public awareness	- General lack of awareness about melanoma risks, prevention, and early detection<br>- Absence of melanoma education in early childhood (e.g., kindergarten), which could be crucial for prevention and timely detection<br>- Relatives of children with melanoma are sometimes blamed for delayed diagnosis, often due to poor public education on the disease<br>- Misconception that melanoma is an “easy” cancer to cure<br>- Confusion about the appropriate doctor (public vs. private) and risk of misdiagnosis<br>-Adapting communication strategies to “speak the same language” as younger people could enhance engagement and awareness
Overdiagnosis	- Financial incentives not to remove moles

	- non incurring into defensive medicine<br>- training of doctors
Underdiagnosis	- Lack of awareness of non-dermatological physicians who see the skin as well<br>- Educate them all during university<br>- good AI-driven early detection tool
Inequality	-Formalize accessibility requirements<br>- Easier requirements to be allowed to study medicine at university<br>- Public observation to track indicators, for instance time to diagnosis<br>- Guidelines<br>- Improved follow up<br>- More experts, more access!
Emotional burden	- Much of the emotional burden comes from the fragmented care pathway that these patients have to walk<br>- The melanoma care journey from diagnosis to follow-up is disjointed<br>- A multidisciplinary team/unit (which should include oncologists, psychologists, and dermatologists with pedagogical training) would be ideal for delivering holistic care<br>- Internal team disputes (at times even in front of the patient) highlight not only the need for better collaboration but also a lack of coordination<br>- Patients face uncertainty about navigating melanoma care regarding choices such as choosing between public or private doctors, understanding costs, and knowing where to seek treatment<br>- Care Navigators could be as a solution to guide patients and coordinate care transitions, a role often seen in the U.S. for wealthier patients<br>- Policy improvements are needed to make Care Navigator services more widely accessible

Table S14. Ethical, Legal and Social Implications of diagnosis and prevention of (ultra)rare diseases: issues found in the literature were presented, and participants of the Focus Group (1) added their inputs. Only those issues on which the participants added something are reported in the results.

**Issues found in the literature**	**Inputs from participants**
Limited knowledge among healthcare professionals	- You cannot expect anyone to know everything: support tools to make knowledge about rare disease accessible<br>- Regular sync<br>- Presence of carer or coordinator during multidisciplinary team meetings<br>- Lack of multi-disciplinarity view and activity about and for the patient<br>- Match making platform to build teams quickly and without bias<br>-And infrastructures, such as biobanks<br>- Research policies: for instance, stipulate sample sharing<br>- Incentivize research for rare diseases
Inequality	- Transgenerational impact<br>- Impact on children of melanoma patients<br>- Little investment in rare diseases in terms of research

Isolation, stigma and discrimination	- Lack of comprehensive plans and policies in place, as well as lack of investment for research fosters a feeling of isolation and discrimination in the patients
Burden of the disease	- Long term financial toxicity, lack of insurability, and education<br>- Care navigator<br>- Lack of pathways
Reimbursement	- Accessible and visible reimbursement policies<br>- Transparency

Table S15. Ethical, Legal and Social Implications in the use of AI-tools for diagnosis in dermatology: issues found in the literature were presented, and participants of the Focus Group (1) added their inputs. Only those issues on which the participants added something are reported in the results.

Issues found in the literature	Inputs from participants
Inaccuracy	- Reliability of an AI-based application, especially when targeting rare diseases such as melanoma in children and the possibility that AI can be trained on such limited data sets<br>- Ensuring the accuracy and reliability of the tool is an important aspect to consider avoiding misdiagnosis and unnecessary anxiety for families<br>- Enrich/correlate raw photos with patient info for better accuracy
Acceptance	- Lack of trust in the tool<br>- The tool must be integrated into a care pathway to be more accepted
Deterioration of doctor-patient relationship	- True, but is that always a good thing?<br>- The doctor/patient interaction is not by any means ideal, or sacrosanct. Google's AMIE AI already outperforms humans
Privacy	- Design the platform privacy first, before using it<br>- Legal framework missing<br>- Data are photos, patient history, patient genetics, location, etc: data sharing and data storage
Responsibility	- AI-based tool is not meant to substitute clinicians, particularly in terms of clinical decision-making. Instead, the app should serve as a platform to improve patient-doctor interaction and facilitate communication with doctors<br>- Dermatologists are still the ones called to avoid "overtreatment", to make decisions<br>- Possible bias in the platform/tool? Yes, and this is why the tool has to be part of a pathway
Job losses	- There are not enough care providers! What is the problem?<br>- May be replaced by new jobs though?
Digital literacy	- AI tools can serve as better replacement for "Dr. Google" and actually improve patient education
Data security	-Legal framework missing<br>- Accountability and transparency
Reimbursement	- Reimbursement is not simply "unclear", it is totally missing!
Responsibility	- Need for high quality control and scrutiny of the tool, giving as an example the serious implications of performing a biopsy or surgical intervention in children based on the findings of the tool

FOCUS SESSION 2

November 19, 2024 - Online

PARTICIPANTS: 5 members + 2 moderators + 1 rapporteur

5 members:

- 1 molecular genetist;
- 1 physician and epidemiologist, director of cancer plan
- 1 telecommunication engineer, data scientist
- 1 physician and professor of medical oncology
- 1 environmental epidemiologist

Participants country of origin: Germany (1), Italy (1), Spain (3).

2 moderators:

- Pietro Refolo, Università Cattolica del Sacro Cuore
- Dario Sacchini, Università Cattolica del Sacro Cuore

1 rapporteur:

- Costanza Raimondi, Università Cattolica del Sacro Cuore

DURATION: 2 hours

Table S16. Ethical, Legal and Social Implications of diagnosis and prevention of childhood melanoma: issues found in the literature were presented, and participants of the Focus Group (2) added their inputs. Only those issues on which the participants added something are reported in the results.

Issues found in the literature	Inputs from participants
Lack of adequate public awareness	- Not only inadequate public awareness, but also low awareness among pediatric oncologists: they are not confident with these patients, as they are keener to treat sarcoma, or leukemia, things are that are more common in that age
Underdiagnosis	- Pediatric oncologists not being confident with these patients poses a problem in terms of underdiagnosis<br>- When diagnosis occurs, it is at advanced stages<br>- To reduce the problem of underdiagnosis, the second opinion of a specialist (pathologist or dermatologist) should be an essential level of assistance, which means also covered by national insurances<br>- Histopathological diagnosis of these lesions is not easy at all: inequalities in access to right diagnosis from a histopathological point of view, because these are rare entities. If you have pathologists that are dedicated to this kind of disease, you have a diagnosis, otherwise, the disease is not easy to be diagnosed
Inequality	- Inequality, based on particular on resources availability and economic status: some people might just not have access to healthcare. This seems a predominant issue<br>- Inequality should also include greater inequality for immigrant patients: in a lot of countries, depending on their legal status, migrants don’t have access to healthcare, and also they don’t know how the system works, so it’s hard to navigate it. They don’t know who do ask

	- This also fits into telemedicine: because of language barriers, how many people who don't speak the language can access telemedicine?<br>- Access of early treatment to prevent recurrence is very low
Privacy	- Privacy in pediatric care must be central
Reimbursement	- Is reimbursement really an issue? One could say that receiving proper care is far more relevant than being reimbursed. However, lack of reimbursement does amplify the social-economic inequality of care, and also inequality across Europe because every country deals with reimbursement in its own way<br>- Reimbursement policies may also have an impact in findings: there is a higher number of cases of childhood melanoma in Sweden and Norway, where you have free access to healthcare. The more you move South, the less cases you have, perhaps due to skin types, but also partially because of access to healthcare, in particular to specialist care

Table S17. Ethical, Legal and Social Implications of diagnosis and prevention of (ultra)rare diseases: issues found in the literature were presented, and participants of the Focus Group (2) added their inputs. Only those issues on which the participants added something are reported in the results.

**Issues found in the literature**	**Inputs from participants**
Limited knowledge among healthcare professionals	- Why is it difficult to be diagnosed? Because it is difficult to find a doctor determined enough to find out the disease that it could be<br>- Do doctors talk to parents about protection from UV radiation, air pollution, and so on? If you have a kid with high risk for genetic predisposition, doctors should educate parents about certain risk factors. Doctors focus only on treatment. The don't see the rest of risk factors, which can have detrimental effects on the person
Inequality	- Inequality and geographic disparities. Certain diseases are more prevalent somewhere and not somewhere else, and this comes with a stigma as well<br>- No funding, no research: why is there no research in rare diseases? Because the patients are few, therefore few people would benefit from drugs/treatments, which means that there is no incentive for big pharma to put any money into it<br>- Environmental justice: your health also depends on where you live, on your education regarding risk factors
Isolation, stigma and discrimination	- Why is it difficult to be diagnosed? Because it is difficult to find a doctor determined enough to find out the disease that it could be<br>- Rare diseases sometimes are associated with a specific population, and if you have this kind of disease, you are connected to a certain kind of population
Burden of the disease	- It is important to have platforms where people who have a rare disease, we have databases, where we can exchange data across Europe to really convince companies to do research on such and such diseases<br>- No funding, no research: why is there no research in rare diseases? Because the patients are few, therefore few people would benefit from drugs/treatments, which means that there is no incentive for big pharma to put any money into it
Informed consent	- Incidental findings is a big issue

Table S18. Ethical, Legal and Social Implications associated with AI-driven tools for prevention and diagnosis in dermatology: issues found in the literature were presented, and participants of the Focus Group (2) added their inputs. Only those issues on which the participants added something are reported in the results.

Issues found in the literature	Inputs from participants
Inaccuracy	- How do we monitor the accuracy of AI, as we should?<br>- How to we keep up with new biases in AI?<br>- AI has to be used carefully. What seems to be most dangerous is to have false positives<br>- AI-tools can be helpful but their input always has to be validated with the assessment of a physician. For now, you cannot rely on AI-tool only<br>- Biased data is very real in AI<br>- Often times AI is said to be an “objective” tool, but we should remember that AI is based on the knowledge of clinicians, so perhaps “objective” is too strong of an adjective<br>- Issues with AI in rare diseases: in order to have machine learning process, you have to put the information in it. This is something that has been done in relatively common diseases, such as adult melanoma, but in rare diseases where does the information (clinical images, histopathological imagines) come from? Are they enough to generate diagnosis?<br>- Challenge of rare diseases: while rare diseases have less data, obviously, here there’s an opportunity to make bigger models trained on bigger data sets adapted to rare diseases. This is important because then you would be able to distribute this
Acceptance	- Empathy is not an issue in AI: it should not be expected from AI, why is it listed among the issues?
Deterioration of the doctor-patient relationship	- Doctors would not be excluded from the process, AI-driven tools must always need validation from a clinician
Privacy	- Privacy concerns raises even more if at some point we want to add genetic information to the AI algorithm. We need to think about what kind of genetic data we want to add: while a patient could not be identified through lesions, it could through genetic information
Responsibility	- AI-tools can be helpful but their input always has to be validated with the assessment of a physician. For now, you cannot rely on AI-tool only<br>- Can AI be accountable for mistakes made? How do we manage such accountability? It is sort of a grey zone, because the human person is the one who accepts the suggestion made by the AI
Job losses	- When talking about ultra-rare diseases, it seems difficult to imagine that AI would have such an impact on the job market. If any, job loss would be minimal or almost. It is important to mention it but not so relevant
Digital literacy	- Digital literacy is extremely relevant, and we could even say that it’s not just digital literacy but literacy overall: the capacity of making a judgment, the capacity of assessing what the AI is telling you<br>- Same thing applies to those who are searching online for interpretation of results. It is a literacy problem<br>- The challenge of digital literacy applies both to patients and to physicians: at times, physicians misunderstand what AI can actually do, and this knowledge gap has to be filled. What is crucial in the use of AI is to understand

	what the problem that needs to be solved is, and what the knowledge that goes into solving it is
Informed consent	- Informed consent: we have to be clear about the role of AI in the process of diagnosis and treatment for this kind of patients in rare and ultra rare diseases. In some cases, it is not easy to explain what can be expected from AI to patients, but it should be done
Reimbursement	- Is the budget for AI-tools part of the assistential budget?
